# Morphological and Morphometric Analysis of the Occipital Condyles and Their Clinical Significance

**DOI:** 10.7759/cureus.95497

**Published:** 2025-10-27

**Authors:** Mehmet C Tatar, Ahmet S Aygündüz, Ekrem Solmaz, Zeliha Fazliogullari

**Affiliations:** 1 Anatomy, Selcuk University Faculty of Medicine, Konya, TUR

**Keywords:** anatomy, condylar canal, dry bone, foramen magnum, occipital condyle, skull base

## Abstract

Background

The occipital bone, located in the human neurocranium, forms the craniovertebral junction and plays a critical role in both structural support and neural protection. The occipital condyle is particularly significant in traumatic events, especially in high-impact injuries such as motorcycle accidents. In this study, we evaluated the anatomy of the occipital condyles, pharyngeal tubercle, and foramen magnum within this trauma-prone region. The goal was to contribute to medical education, improve surgical safety, and provide reference data for clinical applications.

Methodology

A total of 32 dry skulls from a modern Anatolian population were examined in the anatomy laboratory. Linear measurements were taken using a high-precision digital caliper. Parameters measured included the length and maximum and minimum widths of the occipital condyles, the sagittal and transverse diameters of the foramen magnum, and the distances from the pharyngeal tubercle to anatomical landmarks such as the basion, opisthion, and jugular foramen. Morphological classification of the occipital condyles was performed, and the presence of the condylar canal was also recorded. The Kolmogorov-Smirnov test was used to assess normality. Data were expressed as mean ± standard deviation and percentages. The chi-square test was used for categorical variables. For comparisons between two groups, the Student’s t-test (for independent samples) was used under parametric conditions, and the Mann-Whitney U test was applied otherwise. Statistical analysis was performed using SPSS Statistics version 26.0 (IBM Corp., Armonk, NY, USA), with p-values <0.05 considered statistically significant.

Results

The mean sagittal diameter of the foramen magnum was 35.1 ± 2.4 mm, and the transverse diameter was 29.5 ± 2.0 mm. The occipital condyles were classified into nine morphological types. A previously undescribed configuration, provisionally termed the “sausage type,” was observed. Type 3 was the most common on the right side, whereas Type 5 predominated on the left. Bilateral condylar canals were present in 16 skulls and absent in five skulls.

Conclusions

This study identified morphometric variations of potential anthropological and clinical significance. The absence of the condylar canal may reduce risks such as bleeding, atlanto-occipital joint damage, and hypoglossal nerve injury during surgical procedures involving the condylar fossa. Conversely, the absence of the condylar canal may complicate surgery by eliminating an auxiliary venous drainage route to the internal jugular vein. Morphometric variations may influence the choice of surgical approach; for instance, a longer posterior intercondylar distance may provide greater working space during posterolateral approaches. Similarly, in endoscopic endonasal approaches to lesions ventral to the foramen magnum, large occipital condyles may narrow the surgical corridor. Therefore, detailed morphometric knowledge of the occipital condyles is essential for optimizing the safety and effectiveness of craniovertebral surgical interventions.

## Introduction

The occipital condyles (OCs) are paired oval prominences located on the inferior surface of the occipital bone, flanking the foramen magnum (FM) and forming the atlanto-occipital joint with the superior facets of the atlas. Anterior to the condyles lies the pharyngeal tubercle (PT), serving as an attachment site for the pharyngeal raphe, while posteriorly the FM provides passage for the medulla oblongata and vertebral arteries. Together, these structures form the craniovertebral junction (CVJ), a complex anatomical region that is vital for both biomechanical stability and neurosurgical access [1,2].

The occipital bone and its related structures, particularly the OC and FM, play a crucial role in CVJ stability and neurosurgical approaches to the ventral and lateral skull base. Although their anatomy has been described in general terms, population-based morphometric variations can significantly influence both surgical technique and anthropological interpretation. Previous studies have examined these structures in various ethnic groups; however, comprehensive morphometric data specific to the Anatolian population remain limited.

Anatomically, the OCs, which articulate with the vertebral column, are located on the right and left sides of the pars lateralis of the occipital bone. Posterior to the two OCs is the fossa condylaris, which contains the condylar canal (CC). The emisserium vein generally passes through the CC. Another structure in this region is the hypoglossal canal. The anteroposterior diameter of the FM in the occipital bone is wider than the right and left diameters. The topographic point where the anteroposterior diameter intersects the FM anteriorly is called the basion, and the topographic point where it intersects posteriorly is called the opisthion [2,3]. Knowledge of the anatomic characteristics of this skull base region, which includes the FM, OCs, and PT, is important from both surgical and clinical perspectives. Injuries to the region, tumor formations, congenital malformations such as Chiari, cerebrovascular hemorrhage, increased intracranial pressure, cerebral and cerebellar herniations, and various diseases require not only a good knowledge of the anatomy but also morphometric information for surgical interventions. A thorough understanding of the region is crucial for the transcondylar approach procedure, one of the skull base intervention techniques used in neurosurgery [4]. Furthermore, knowledge of the anatomy of the region is important for skull base surgery and procedures, including lateral and infratemporal approaches and access to lesions [5].

Various surgical procedures are used to plan operations on the skull base region and address the disease. Pre-procedure planning determines the approach to be used. Transpetrosal, transcondylar suboccipital, and extreme lateral approaches are used to access the region for skull base operations. However, these surgical procedures carry a high risk of neurological deficits. Region-related morphometry and morphology influence surgical interventions [6]. Morphological and morphometric analyses of human bones are also widely utilized in forensic medicine, anthropology, and archaeological assessments [7].

The occipital bone and its related structures, particularly the OCs and FM, play a crucial role in CVJ stability and neurosurgical approaches to the ventral and lateral skull base. Although their anatomy has been described in general terms, population-based morphometric variations can significantly influence both surgical technique and anthropological interpretation. Previous studies have examined these structures in various ethnic groups; however, comprehensive morphometric data specific to the Anatolian population remain limited.

Considering the growing importance of individualized surgical planning, especially for transcondylar, far-lateral, and endonasal approaches, defining region-specific morphometric parameters is of high clinical relevance. Therefore, the present study aims to provide detailed morphometric and morphological data of the OCs in Anatolian skulls and interpret their clinical and surgical implications within the context of the existing literature.

## Materials and methods

**Figure 1:**
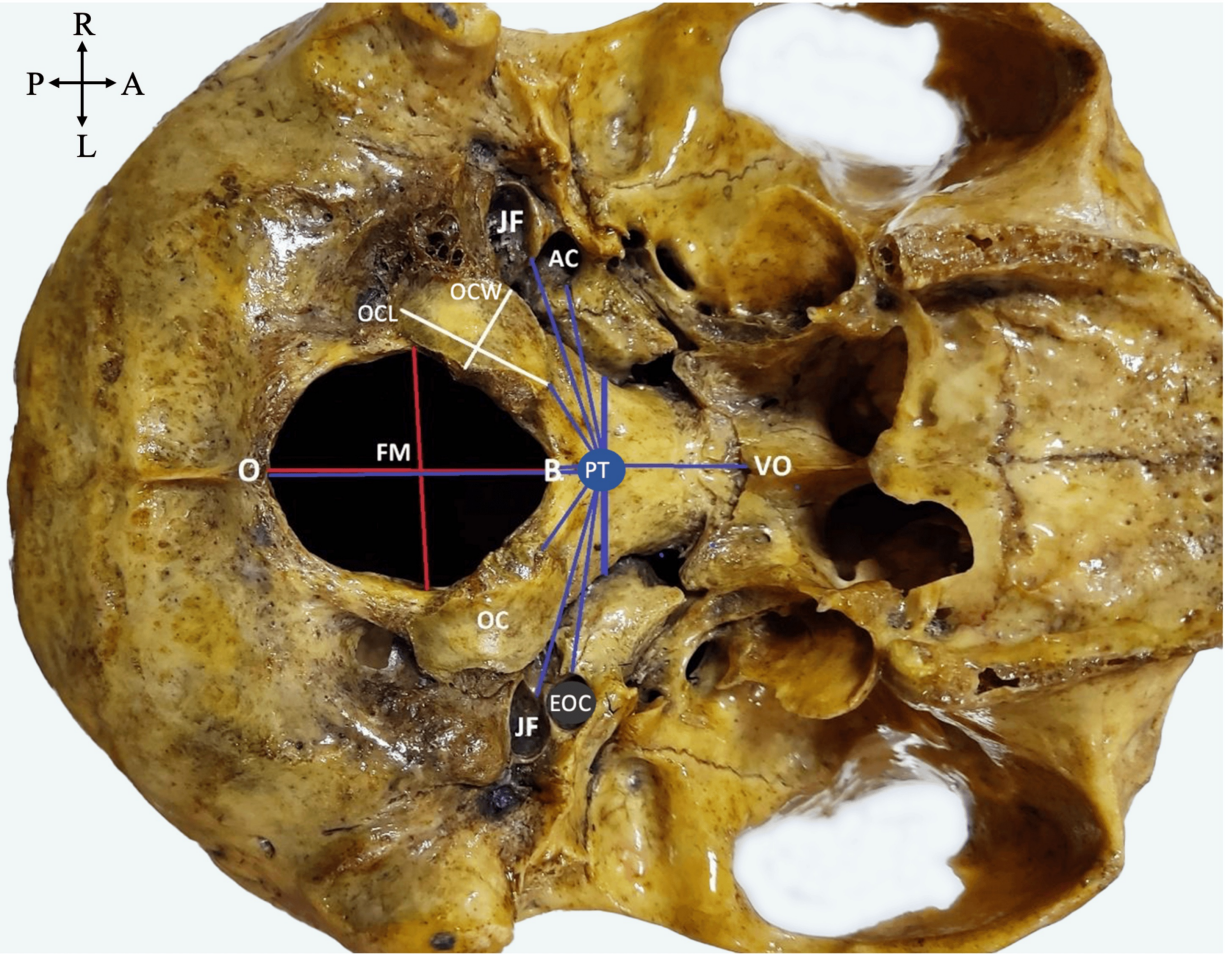
Measurement points (inferior view of the skull base). FM: foramen magnum; OC: occipital condyle; PT: pharyngeal tubercle; O: opisthion; B: basion; VO: vomer; JF: jugular foramen; red line between O and B: anteroposterior diameter of FM (FMAP); red line between the OCs: transverse diameter of the FM (FMT); EOC: external opening of the carotid canal; thick blue line: length of the basilar part measured from the PT (PTBP); long white line: OC length (OCL), OC width (OCW); P: posterior; A: anterior; R: right; L: left

**Figure 2:**
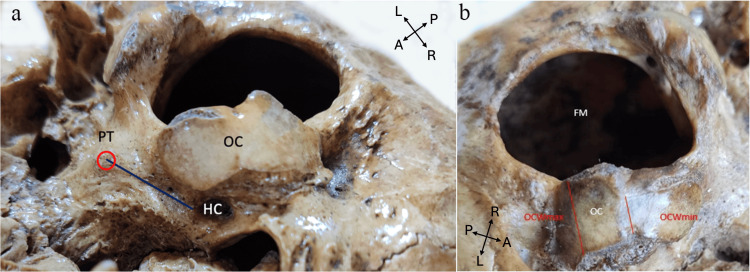
Measurement locations (inferior and lateral views of the skull base). (a) PT: pharyngeal tubercle (red circle); OC: occipital condyle; HC: hypoglossal canal. Blue line: distance of the PT to the external opening of HC (PTEHC). (b) OC: occipital condyle; FM: foramen magnum; OCWmax: maximum width of the OC; OCWmin: minimum width of OC; P: posterior; A: anterior; R: right; L: left

**Figure 3:**
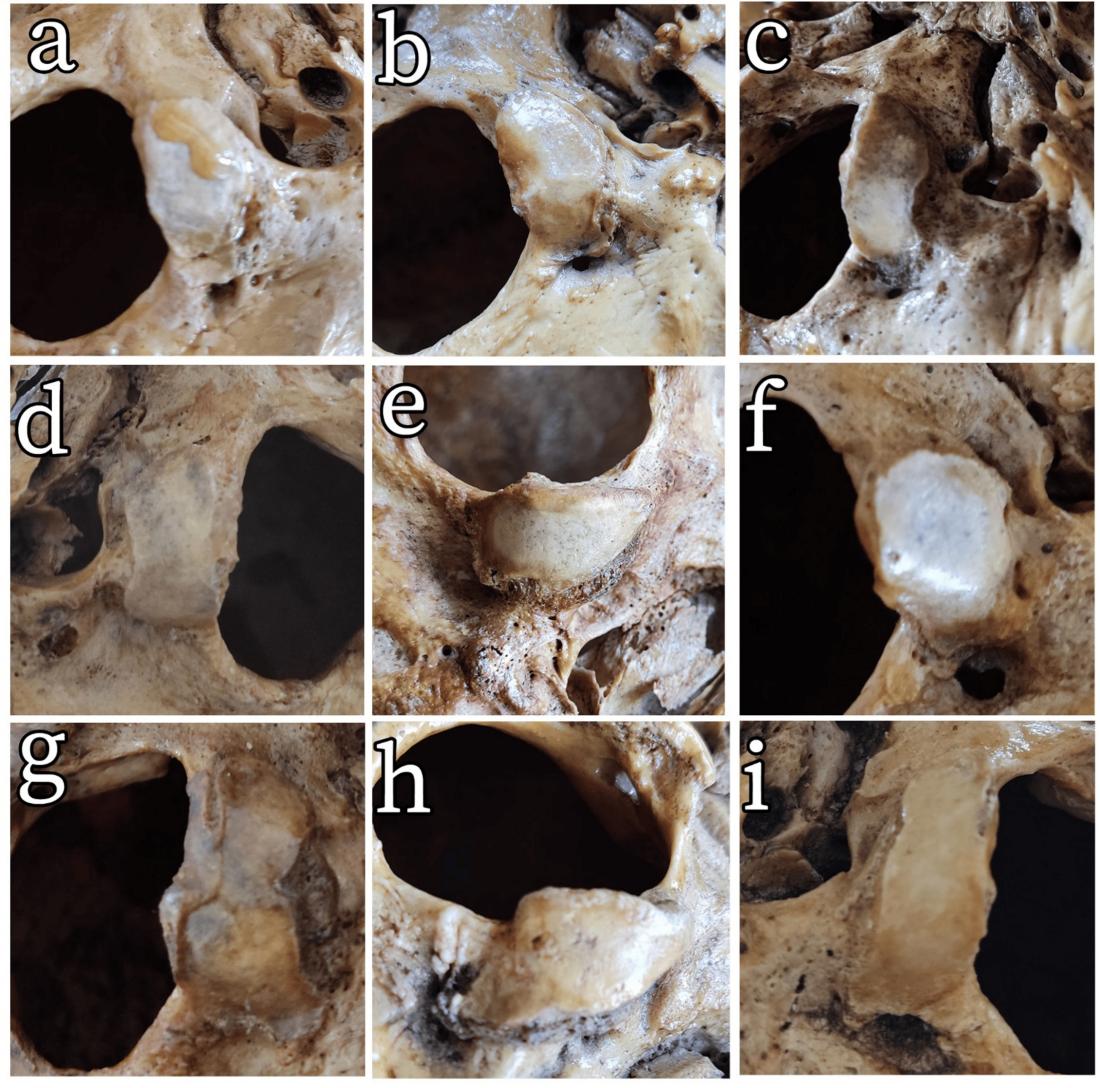
Occipital condyle types. (a) Oval-like. (b) Kidney-like. (c) S-like. (d) Eight-like. (e) Triangle. (f) Ring-like. (g) Two-portioned. (h) Deformed. (i) Sausage-like.

**Figure 4:**
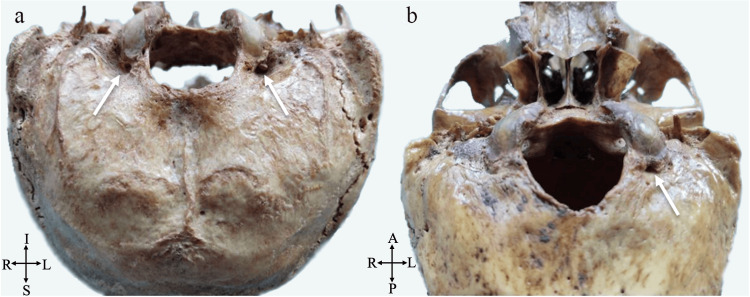
Condylar canal (white arrow) locations. (a) Posterior view of the bilateral condylar canal (S: superior, I: inferior, R: right, L: left). (b) Posteroinferior view of the unilateral (left) condylar canal (P: posterior, A: anterior, R: right, L: left).

Ethical approval for this study was obtained from the Selçuk University Faculty of Medicine Local Ethics Committee (date: August 01, 2023, meeting number: 2023/15, decision number: 2023/371). A total of 32 dry human skull bones belonging to the Anatolian population in the Anatomy Laboratory of Selçuk University Faculty of Medicine were examined. In some parameters of the classification and measurements, the total number did not reach 32 due to bone deformities that prevented proper classification or measurement on the relevant sides. The age, sex, and clinical status of the skulls were unknown. Anatomical structures were measured using a digital caliper (Insize 1108/Suzhou, People’s Republic of China), with a measuring range of 0-150 mm and a sensitivity of 0.03 mm. The measurements were conducted twice by the same researcher with a 15-day gap and assessed based on their averages. The intraclass correlation coefficient was calculated to assess intraobserver reliability, demonstrating excellent agreement with values exceeding 0.90 for all measurements (95% confidence interval = 0.88-0.97). The Kolmogorov-Smirnov test was used to check the suitability of the data for normal distribution. The data obtained were presented as mean ± standard deviation and percentages, and the chi-square test was used for the evaluation of categorical data. For comparisons between two groups, Student’s t-test (t-test for independent groups) was used when parametric conditions were met, and the Mann-Whitney U test was used when they were not. All statistical analyses were performed using SPSS Statistics version 26.0 (IBM Corp., Armonk, NY, USA), with statistical significance set at p-values <0.05. The morphometric parameters evaluated in our study are shown in Table 1. We evaluated morphological parameters (Figures 1, 2), the shape of the condyle (Figure 3), the location of the internal opening of the canal relative to the condyle, the location of the external opening of the canal relative to the condyle, typing based on the presence of a septum or notch in the canal, and the presence of the CC (Figure 4).

**Table 1:** List of morphometric parameters measured.

Morphometric parameters	Abbreviation
The anteroposterior diameter of the foramen magnum	FMAP
The transverse diameter of the foramen magnum	FMT
The occipital condyle length	OCL
The maximum width of the occipital condyle	OCWmax
The minimum width of the occipital condyle	OCWmin
The anterior intercondylar distance	AID
The posterior intercondylar distance	PID
The distance of the pharyngeal tubercle to the basion	PTB
The distance of the pharyngeal tubercle to the opisthion	PTO
The distance of the pharyngeal tubercle to the external opening of the carotid canal	PTEOC
The distance of the pharyngeal tubercle to the jugular foramen	PTJF
The distance of the pharyngeal tubercle to the vomer	PTV
The distance of the pharyngeal tubercle to the anterior edge of the occipital condyle	PTAOC
The length of the basilar part measured from the pharyngeal tubercle	PTBP
The distance of the pharyngeal tubercle to the external opening of the hypoglossal canal	PTEHC

## Results

Morphometric results

Morphometric measurements of the foramen magnum are shown in Table 2. A correlation was found between the transverse diameter of FM (FMT) and the anteroposterior diameter of FM (FMAP).

**Table 2:** Anteroposterior and transverse diameter measurements of the foramen magnum (mm). FMAP: anteroposterior diameter of the foramen magnum; FMT: transverse diameter of the foramen magnum

Parameter	n (%)	Mean ± SD	Minimum	Maximum
FMAP	30 (93.7%)	35.1 ± 2.4	30.3	40.4
FMT	32 (100%)	29.5 ± 2.0	26.3	33.8

The OC length (OCL), maximum width of the OC (OCWmax), anterior intercondylar distance (AID), and posterior intercondylar distance (PID) were measured. Additionally, the minimum width of the OC (OCWmin) was measured for Types d and g. No significant difference was found between the right and left sides except for OCWmax (p = 0.007). The mean values ​​of OCL, OCWmax, OCWmin, AID, and PID were found to be 24.53 ± 3.04 mm, 11.94 ± 1.12 mm, 7.99 ± 1.75 mm, 19.79 ± 2.67 mm, and 42 ± 3.57 mm, respectively (Table 3).

**Table 3:** Morphometric measurements of the OC (mm). OC: occipital condyle; OCL: OC length; OCWmax: maximum width of the OC; OCWmin: minimum width of the OC; AID: anterior intercondylar distance; PID: posterior intercondylar distance. Type d: eight-like condyle, Type g: two-portioned condyle

Parameter/Type	Left		Right		Total	
-	n (%)	Mean ± SD	n (%)	Mean ± SD	n (%)	Mean + SD
OCL	28 (87.5%)	24.2 ± 3.1	29 (90.6%)	24.8 ± 3.0	57 (89%)	24.5 ± 3.0
OCWmax	28 (87.5%)	12.3 ± 1.0	30 (93.7%)	11.5 ± 1.0	58 (90.6%)	11.9 ± 1.1
OCWmin (Types d and g)	4 (12.5%)	7.9 ± 2.0	7 (21.8%)	8.0 ± 1.7	11 (17.1%)	8 ± 1.7
AID	-	-	-	-	27 (84.3%)	19.8 ± 2.6
PID	-	-	-	-	32 (100%)	42.0 ± 3.5

The distance of the PT to the external opening of the carotid canal (PTEOC), distance of PT to the jugular foramen (PTJF), distance of the PT to the anterior edge of the OC (PTAOC), and distance of PT to the external opening of the hypoglossal canal (PTEHC) were measured bilaterally, while the distance of PT to vomer (PTV), ength of the basilar part measured from the PT (PTBP), and distance of PT to basion (PTB) were measured unilaterally (Table 4). No significant difference was found between the right and left sides (p > 0.05).

**Table 4:** Parameters of the PT (mm). PT: pharyngeal tubercle; PTEOC: distance of PT to the external opening of the carotid canal; PTJF: distance of PT to the jugular foramen; PTAOC: distance of PT to the anterior edge of the occipital condyle; PTV: distance of PT to the vomer; PTO: distance of PT to opisthion; PTB: distance of PT to basion; PTPB: length of the basilar part measured from the PT; PTEHC: distance of PT to the external opening of the hypoglossal canal

Parameter	n (%)	Mean ± SD	Minimum	Maximum
PTEOC	57 (89%)	27.3 ± 2.5	22.4	35.0
PTJF	58 (90.6%)	25.2 ± 2.0	19.2	30.2
PTAOC	57 (89%)	15.6 ± 1.9	12.5	20.0
PTV	29 (90.6%)	16.4 ± 2.0	11.8	22.5
PTO	30 (93.7%)	45.4 ± 3.0	39.8	51.8
PTB	32 (100%)	11.0 ± 1.5	8.1	14.2
PTPB	32 (100%)	29.7 ± 4.5	18.6	40.0
PTEHC	64 (100%)	19.7 ± 1.6	16.2	24.3

Morphological results

The OC was divided into nine classes based on its shape (Figure 3). Type 3 was the most common on the right (seven cases, 23.3%). Type 5 was the most common on the left (seven cases, 25%). Type 8 was the least common condyle type on the right (one case, 3.3%). Type 8 (one case, 3.6%) and Type 7 (one case, 3.6%) were the least common condyle types on the left (Table 5). There were differences between the right and left condyles in 15 of 28 skulls. The chi-square test revealed no significant difference between the right and left sides (p > 0.05). No condyle was found in the median (third) of 32 skulls. When the OC types were evaluated, no significant difference was observed between the right and left sides (p = 0.760).

**Table 5:** Classification of the occipital condyles types.

Subimages in Figure 3	Shapes	Left		Right		Total	
-	-	n	%	n	%	n	%
a	Oval-like	4	14.3	4	13.3	8	13.8
b	Kidney-like	3	10.7	3	10.0	6	10.3
c	S-like	4	14.3	7	23.3	11	19.0
d	Eight-like	6	21.4	4	13.3	10	17.2
e	Triangle	7	25.0	4	13.3	11	19.0
f	Ring-like	2	7.1	2	6.7	4	6.9
g	Two-portioned	1	3.6	3	10.0	4	6.9
h	Deformed	1	3.6	1	3.3	2	3.4
i	Sausage like	0	0.0	2	6.7	2	3.4
-	Total	28	100.0	30	100.0	58	100.0

The presence of a CC in the condylar fossa was evaluated bilaterally. Out of 32 skulls, CC was observed on the left side in 22 (68.75%) cases and on the right side in 19 (61.2%) cases. A double CC was observed in one skull. In five skulls, CC was absent bilaterally, whereas in 16 skulls, CC was present bilaterally. In four skulls, CC was absent on the left but present on the right. In five skulls, CC was absent on the right but present on the left. CC was present unilaterally in nine skulls (Figure 4).

## Discussion

In the present study, when the OC types and morphometric parameters were evaluated, no significant side differences were found except for OCWmax (p = 0.007). OC morphometric measurements may influence transcondylar surgical technique. Our data are from an Anatolian population, and evaluations should be clinically specific to each case. Morphometric data are also supportive. The mean OCL, OCWmax, OCWmin, AID, and PID values were consistent with previous anatomical reports. Similarly, no right-left asymmetry was detected in PT-based measurements. The OC was classified into nine morphological types. Type 3 was most frequent on the right and Type 5 on the left, while Types 7 and 8 were the least common. These morphological features may influence nail placement procedures in skull base surgery. Although variations between sides were observed in several skulls, no statistically significant asymmetry was found (p > 0.05). A CC was identified in 68.7% of left and 61.2% of right sides, being bilateral in half of the specimens and absent in five skulls. Such variations are likely to occur, and preoperative assessment is clinically appropriate.

The relationship between the OC, hypoglossal canal, and jugular tubercle is important for far lateral approaches [8]. Therefore, the location of the canal relative to the condyle can help predict its course and may be advantageous in surgical procedures involving drilling of the condyle, such as the transcondylar approach. Considering that the canal rarely runs posterior to the condyle, it has been suggested that surgical procedures should be performed posteriorly [9]. Wang et al. [10] noted that the hypoglossal canal could serve as a landmark in endoscopic nasal approaches to FM, further highlighting the significance of its location. A different clinical investigation conducted a biomechanical analysis, revealing that removal of over 75% of the condyle did not correlate with clinically significant CVJ instability [11].

The FM is a crucial structure for skull base surgery in clinical situations because it allows the passage of important vessels and nerves, particularly the spinal cord. Potential complications can lead to significant morbidity and mortality [10]. Therefore, a thorough understanding of the anatomy of the FM may affect survival rates.

In our study, FMAP (35.19 ± 2.42 mm) and FMT (29.58 ± 2.01 mm) measurements were consistent with previous studies. These values ​​were found to be 35.05 ± 2.57 and 30.19 ± 2.69 by Lyrtzis et al. [12], 34.8 mm and 29.6 mm by Kızılkanat et al. [13], 34.7 mm by Naderi et al. [14], 33.3 mm and 27.9 mm by Muthukumar et al. [15], 35.53 ± 3.06 and 30.31 ± 2.79 mm by Natsis et al. [16], 36.6 mm by Bozbuğa et al. [17], and 31 ± 2.4 to 25.2 ± 2.4 mm by Chethan et al. [18]. In their studies conducted on the Indian population, Muthukumar et al. [15] and Chethan et al. [18] found significantly smaller values ​​compared to other studies. Many researchers reported larger FMAP and FMT in men than in women. These differences may be due to factors such as race, age, and environment. Gruber et al. [19] stated that FMAP size is similar in humans regardless of ethnic group and cannot be used as a means of identifying populations. Comparisons made without using the same age, sex, measurement technique, or clinical conditions may yield different results. Differences in the literature regarding measurements of the FM may be due to study groups. They also noted that the differences found between populations in previous studies may be due to insufficient samples and a lack of detailed statistical analysis.

In our study, a strong, positive correlation was found between FMAP and FMT values. Reaching the internal opening of the canal in patients with a large FM may require traversing more soft tissue and resecting more bone [15]. Additionally, it has been noted that a correlation between FMT and FMAP could be useful for anthropologists and forensic medicine specialists [19]. Therefore, even if the skull base is damaged, other parameters can still be estimated.

The OC forms the lateral border of the FM, the inferior wall of the hypoglossal canal, and the articular surface of the atlanto-occipital joint. Many different methods have been used in the literature when classifying the OC according to its shape [14]. Because of high subjectivity, results may differ significantly even when the same method is applied. In our study, condyles were classified into nine types, with S-like and triangle-type OC being the most common. The two condyles that did not fit the classification of Naderi et al. [14] were classified as sausage-like (Figure 3, Table 5). A previously undescribed morphology, tentatively termed the “sausage type,” was observed. It shares features with Type I (oval-like) in Naderi et al.’s classification, but differs by its elongated, smooth, cylindrical contour and minimal curvature. While Naderi et al. [14] reported the oval-like condyle as the most common type (50%) in their own classification, Kalthur et al. [20] identified eight-like (22.5%) and oval-like (22.5%) types as the most common. Natsis et al. [16] observed S-like condyles most frequently in both sexes (right: 33.1%, left: 28.3%) and, unlike other studies, reported condylar asymmetry between the right and left sides. Bozbuğa et al. [17] used a different classification system. Naderi et al. [14] stated that these classifications are complex and have limited clinical relevance. Gapert et al. [21] detected OC dimorphism between men and women and suggested that this may be due to genetic-epigenetic factors. This may not only explain sex-related differences but could also underlie inter-population variations in OC morphology. Researchers’ interpretations of these types are the reason for these differences.

As the amount of condyle resected is closely related to atlanto-ocipital joint instability [8,10], ring-like condyles cause more atlanto-ocipital joint instability than other types when the same amount of bone resection is performed due to their smaller joint surfaces. When preservation of the CVJ is intended, an approach from the condylar fossa can be considered. It is more advantageous as it provides access with a clearer visualization and allows for less resection. Oval-type condyles have been reported to have higher surgical success due to their larger surface area [22]. In contrast, triangle-type, deformed-type, and kidney-like-type condyles have been reported to require more condylectomy and may increase the risk of complications [10,14]. Morphologically, the shape of OC may affect joint movements and may play a role in determining the surgical technique for skull base procedures.

The OCL, OCWmax, OCWmin, AID, and PID ends of the right and left condyles were measured (Table 3). No significant difference was found in the bilateral measurements except for OCWmax (p < 0.05). Our measurements were consistent with previous studies [12,17,20]. Our findings were close to the measurements of Ozer et al. [22] but greater than the values reported by Bayat et al. [23] (OCL = 19.35 ± 3.41 mm, OCW = 9.31 ± 1.91 mm, AID = 15.39 ± 7.99 mm, PID = 35.60 ± 8.4 mm). Bayat et al. [23] used bones of unknown age in their study on the Iranian population. Gruber et al. [24] noted that as the base of the occipital bone continues to develop under the influence of genetic factors until the age of 10, its dimensions vary. Therefore, Bayat et al. [23] may have used pediatric bones (Table 6). Unlike other studies, the OCWmin distance was measured, allowing for a more objective assessment and clearer morphological classification of the condyle.

**Table 6:** Comparison of OC measurements (mm). OC: occipital condyle; OCL: OC length; OCW: OC width; AID: anterior intercondylar distance; PID: posterior intercondylar distance; M: male; F: female; R: right; L: left; ds: dry skull

Study	n	OCL	OCW	AID	PID
Bozbuga et al. (1999) [17]	76 ds 8 cadaver	-	-	22.8	30.2
Naderi et al. (2005) [14]	202 ds	23.6	10.5	21±2.8	41.6±2.9
Muthukumar et al. (2005) [15]	50 ds	26.6	14.7	-	-
Kizilkanat et al. (2006) [13]	59 ds	24.5	13.1	22.6	44.2
Gapert et al. (2009) [21]	158 ds	24.9 ± 2.5 (M, R); 23.3 ± 2.2 (F, R); 25.1 ± 2.5 (M, L); 23.7 ± 2.4 (F, L)	12.0 ± 1.4 (M, R); 11.4 ± 1.2 (F, R); 12.0 ± 1.6 (M, L); 11.5 ± 1.1 (F, L)	51.2 ± 2.9 (M); 48.6 ± 3.1 (F)	36.8 ± 3.1 (M); 35.1 ± 3.0 (F)
Özer et al. (2011) [22]	144 ds	23.9 ± 3.4 (R); 24 ± 3.3 (L)	11.9 ± 2.3 (R); 10.7 ± 2.3 (L)	20.9 ± 3.6	43.1 ± 4.0
Natsis et al. (2013) [16]	143 ds	25.6 ± 2.9 (R); 25.6 ± 2.7 (L)	13.0 ± 1.9 (R); 13.0 ± 1.9 (L)	19.3 ± 3.2	51.6 ± 5.0
Kalthur et al. (2014) [20]	71 ds	22 ± 2	11 ± 2	-	-
Bayat et al. (2014) [23]	50 ds	19.3 ± 3.4	9.3 ± 1.9	15.3 ± 7.9	35.6 ± 8.4
Ilhan et al. (2017) [25]	100 ds	23.47 ± 2.44	11.4 ± 1.4	22.4 ± 2.9	41.5 ± 3.7
Our study (2025)	32 ds	24.5 ± 3.0	11.9 ± 1.1	19.8 ± 2.6	42 ± 3.5

The size of the OCs is crucial for neck stability, the functionality of neurovascular structures, the craniocervical junction, and the planning and execution of surgical interventions [15,17]. The size of the condyles can influence the choice of surgical approach, particularly in the removal of tumors in the craniocervical region. While larger condyles may be advantageous because they allow for more bone resection during surgery, they may also be disadvantageous because they may not provide optimal visibility [20]. Clinical effects of morphometric measurements and morphology will contribute to the determination of surgical techniques. A longer PID distance may provide more free space in the posterolateral approach [22]. Similarly, in the endonasal endoscopic approach used for tumors located ventral to the FM, the condyles have been reported to narrow the surgical pathway [10]. Therefore, a longer AID distance may provide a wider corridor and thus increase surgical success. Consequently, the size and morphology of the OCs should be considered in clinical practice.

On the lower surface of the basilar pars of the occipital bone, there is a protrusion called the PT, to which the pharynx raphe attaches [2]. In our study, we measured the distances of this protrusion to some structures. Consistent with previous studies, no right-left asymmetry was observed (p > 0.05). However, the PTPB value was significantly higher than that reported in previous studies, the PTEOC was bilaterally smaller, and other parameters were similar (Table 7). We emphasize that a greater PTBP distance may indicate an elongated clival or basilar segment, potentially affecting the surgical trajectory and working corridor in endoscopic endonasal approaches to the ventral FM. This additional space could influence the depth perception, angle of approach, and instrument maneuverability during skull base procedures. Krmpotić-Nemanić et al. [1] observed that the size of the basilar pars increases with age until adulthood and remains relatively unchanged thereafter. This may explain why Ceri et al. [26] and Krmpotić-Nemanić et al. [1] reported different results. However, in our study, we obtained large measurements that were not related to age. This may be due to differences in the studied population, the measurement method, or unknown clinical conditions.

**Table 7:** Comparison of PT measurements (mm). PT: pharyngeal tubercle; PTB: distance of PT to basion; PTO: distance of PT to opisthion; PTV: distance of PT to vomer; PTBP: length of the basilar part measured from the PT; PTAOC: distance of PT to the anterior edge of the occipital condyle; PTEOC: distance of PT to the external opening of the carotid canal; PTJF: distance of PT to the jugular foramen; PTEHC: distance of PT to the external opening of the hypoglossal canal; R: right; L: left

Parameters	Krmpotić-Nemanić et al. (2006) [1]	Ceri et al. (2021) [26]	Our study (2025)
PTB	8.6 ± 2	11.9 ± 1.2	11.0 ± 1.5
PTO	-	44.6 ± 2.8	45.4 ± 3.0
PTV	17.2 ± 3.8	18.5 ± 1.5	16.4 ± 2.0
PTBP	26.8 ± 5.5	24.1 ± 2.7	29.7 ± 4.5
PTAOC-R	-	15.3 ± 1.6	15.6 ± 2.0
PTAOC-L	-	15.2 ± 1.4	15.7 ± 1.7
PTEOC-R	-	31.6 ± 2.7	27.3 ± 2.7
PTEOC-L	-	30.2 ± 1.4	27.4 ± 2.3
PTJF-R	-	-	25.4 ± 2.0
PTJF-L	-	-	25.0 ± 2.1
PTEHC-R	-	-	19.4 ± 1.5
PTEHC-L	-	-	20.0 ± 1.6
Specimen (n)	16	60	64

The distances of the PT to different anatomical structures may play an important role in the stability of the craniocervical region, neurological and vascular surgical procedures, endoscopic approaches, and surgical planning [27]. Fang et al. [27] measured the distance of the PT to the anterior border of the external opening of the CC (EOC), rather than its closest distance to the EOC. They obtained smaller results than expected (25.2 ± 3.2 mm). They stated that the PT can be a reliable landmark in identifying the location of the internal carotid artery. These findings should be considered in clinical practice to achieve better outcomes in patient treatment and care and to ensure the safety of surgical interventions.

The CC is a large canal containing the largest emissary vein, which connects the suboccipital plexus to the sigmoid sinus [3]. In our study, the presence of CC was evaluated bilaterally. CC was present in 25 of 32 skulls (78%) and 41 of 64 sides (64%). Ginsberg [28] evaluated CT images in his study and detected CC in 81% of patients and 73.5% of dry skulls. Similar to our study, Ginsberg [28] did not detect right-left dimorphism.

Bozbuga et al. [17] observed CC in 89% of 76 skulls. Bayat et al. [23] observed CC in 60% of samples in the Iranian population, which is less than that reported in other studies. Similar to Bayat et al. [23], Muthukumar et al. [15] found CC in 60% of the samples, more frequently on the right side. Matsushima et al. [29] found CC in CC in 19 of 25 (76%) skulls (Table 8).

**Table 8:** Presence of CC in studies. CC: condylar canal; Presence: presence of CC on at least one side; CC absence: absence of bilateral CC; ds: dry skull

Presence/Study	Ginsberg (1994) [28]	Bozbuga et al. (1999) [17]	Muthukumar et al. (2005) [15]	Bayat et al. (2014) [23]	Matsushima et al. (2014) [29]	Our Study (2025)
CC absence (-)	29 (19%)	8 (11%)	20 (40%)	20 (40%)	6 (24%)	7 (22%)
CC presence (+)	94 (81%)	68 (89%)	30 (60%)	30 (60%)	19 (76%)	25 (78%)
n (%)	123 (100%) CT	76 (100%) ds	50 (100%) ds	50 (100%) ds	25 (100%) ds	32 (100%) ds

Small foramina and canaliculi were observed in some bones in the condylar fossa, but these were not considered CC. While CC was detected in most samples in previous studies, different rates were obtained even when studied in the same population. This may be due to the size of the canal canaliculus, which is considered CC. As a result, CC is a normal formation commonly observed in humans.

When access to the FM and adjacent lesions is desired and the atlanto-occipital joint needs to be preserved, a surgical approach through the condylar fossa may be necessary [8,29]. The absence of the CC may protect against disadvantages such as bleeding, atlanto-occipital joint damage, and hypoglossal nerve damage during surgical procedures performed on the condylar fossa. However, the absence of the CC may complicate surgical procedures due to the lack of an alternative drainage pathway to the internal jugular vein [30]. In the diagnostic methods of CT angiography and MR angiography, the absence of contrast enhancement posterior to the condyle in the absence of the CC should not always be interpreted as a pathological condition [28]. The CC and condylar emissary vein are important because they form an alternative drainage pathway together with other emissary veins when blood flow from the sigmoid sinus to the internal jugular vein is interrupted [30]. Additionally, it should be kept in mind that the absence of CC may lead to differences in venous blood flow, potentially resulting in a different clinical presentation in cases of thromboembolism and cancers that metastasize via the venous pathways. The present findings have direct implications for neurosurgical approaches to the CVJ. The observed mean values of the OC and intercondylar distances suggest potential variation in the surgical corridor width for transcondylar and far-lateral approaches, which rely heavily on condylar morphology and orientation. A shorter AID, for instance, may narrow the operative field and increase the need for condylar resection to achieve adequate exposure, whereas wider condyles could influence screw placement trajectories. Additionally, population-specific morphometric differences, such as those identified in this Anatolian sample, highlight the importance of individualized preoperative planning based on three-dimensional imaging. These findings, therefore, contribute both anatomically and clinically by refining the morphometric reference data used in skull base surgery.

Limitations of our study include that the age, sex, and clinical status of the skulls were unknown. Furthermore, the sample size was relatively small compared to other studies. Additionally, demographic information such as sex and age of the skulls was unavailable due to the archival nature of the sample, preventing demographic comparisons. The relatively small sample size may also limit the statistical power and generalizability of the findings to other populations. Future studies with larger, demographically controlled, and radiologically confirmed samples are recommended.

## Conclusions

In our study, we performed measurements and morphological assessments of the skull base. While the findings were generally similar to previous studies, some different measurements and variations were identified that may be anthropometrically and clinically significant. This study provides the first comprehensive morphometric and morphological analysis of OCs in an Anatolian population, contributing novel population-specific reference data. The findings have direct clinical relevance for surgical procedures involving the CVJ, particularly in optimizing the extent of condylar resection and improving preoperative planning for transcondylar and far-lateral approaches. The morphometric variations observed also hold anthropological value in understanding regional skeletal diversity. Future research integrating radiological imaging and demographic variables across larger and more diverse populations is recommended to validate these results and further refine their clinical applicability.
